# Editorial: Queer(ing) age(ing)

**DOI:** 10.3389/fsoc.2024.1435561

**Published:** 2024-07-09

**Authors:** Miranda Leontowitsch, Dana Rosenfeld, Ralf Lottmann, Jesus Ramirez-Valles

**Affiliations:** ^1^Youth and Social Services, Independent Researcher, Frankfurt, Germany; ^2^Visiting Scholar, University of Westminster, London, United Kingdom; ^3^Social Work, Health and Media, Magdeburg Stendal University of Applied Sciences, Magdeburg, Saxony-Anhalt, Germany; ^4^Department of Medicine, University of California, San Francisco, San Francisco, CA, United States

**Keywords:** aging, queer theory, gerontology, LGBTQI^*^, queer aging, heteronormative aging, structure and agency

Queer aging is a collective term with several meanings: It is used to highlight how LGBTQI^*^ identities, communities, and aging are structural categories of difference, how they interact with other categories of difference, and these interactions' implications for social inequalities (e.g., Lottmann, 2021). By examining LGBTQI^*^ persons' lived experiences of growing older and of later life, queer aging increases these persons' visibility and contributes to more differentiated images of aging (e.g., Rosenfeld, 2010; Ramirez-Valles, 2016). Finally, queer aging is a theoretical perspective that combines queer theory with critical gerontology to reveal and critique gerontology's reliance on approaches rooted in heteronormativity, life course theory, and successful aging (e.g., Sandberg, 2008; Sandberg and Marshall, 2017). Queer approaches to aging enable new ways of disrupting binaries in aging studies—successful/failed, healthy/pathological, lonely/happy—and thereby deconstructing heteronormativity and ableism in mainstream gerontology (Sandberg and Marshall, 2017).

The term “queer” has its roots in a post lesbian and gay liberation movement political activism that reclaimed the term queer from its derogative meaning and usage while retaining the term's reference to otherness and transgression (Butler, 1990; Sedgwick, 1990). Arising from anti-discriminatory activism, queer coalitions formed in the 1990's in the United States broadened to include the realities of bi, trans, inter-sex and non-gender persons and queers of color, thereby integrating the connection of race and sexuality into queer theorizing (Rauchut, 2008). Despite building on the gains made by the lesbian and gay movement(s), queer theory is broader and includes several theoretical schools that come together in a theoretical and political process. These theoretical strands include post-structuralism, deconstructionism, feminism, Lacanian psychoanalysis, and Foucauldian biopolitics. At its core, queer theory questions knowledge production by questioning (“queering”) existing concepts as well as the frameworks, institutions and practices that produce knowledge (Hark, 2001). Queer theory has been used to challenge normative notions of self, identity, temporality, spaces, class, and the nature of being in the world. But it also critiques the construction of unitary gay identities, and a gay identity politics, that reify a wide range of fluid sexualities and genders.

Several parallels between age, gender and sexuality lend themselves to combining critical gerontology with queer theory: sexuality and age are not static and “natural,” but change over the life course; performing a coherent older, gendered/sexual self is crucial for being culturally intelligible; and the embodiment of age, gender and sexuality is an ongoing negotiation of corporeality, social construction, and discourse (Port, 2012; Sandberg and Marshall, 2017). By combining critical gerontology and queer theory, it is possible to interrogate the normativities and structures that shape social perspectives on and experiences of aging and later life, including heteronormative structures within gerontology itself.

Queer aging is still a relatively new and uncharted research terrain. Following Leontowitsch and Werny (2021), we connect the work by older lesbians in the women's rights movement (e.g., Barbara Macdonald, Cynthia Rich) of the 1980's and by critical gerontologists studying social inequalities (e.g., Toni Calasanti). Queer aging's innovative potential lies in making visible the lives of marginalized older people, and in incorporating their experiences and subversive practices as new narratives into existing understandings of aging (Fabbre, 2015). This can contribute to more differentiated and less stereotypical images of aging. Queer aging also questions heteronormative assumptions about later-life social support and care and how they are engrained in social policy. However, it is important to note that queer aging (as many other queer theoretical perspectives) come from Western (white) scholars and communities. Current work has not fully engaged with theories and scholars on racism and colonialism to better understand how aging, outside normative scripts, takes place.

This Research Topic brings together four papers that illustrate the potential of queering aging, both in terms of the experiences of LGBTQI^*^ aging, and unpacking knowledge about understanding later life at large. King and Hall examine how queer theory can critically engage with several different aspects of LGBTQI^*^ aging (i.e., chronology, cognition, and “frailty” and vulnerability). It can challenge how normativity is manifested through these aspects and further sociological knowledge concerning the complex and often contradictory implications that queer aging has for understanding later life.

Keller applies a queer perspective to notions of successful aging and the experiences of people aging with dementia. Drawing on qualitative interviews with people living with dementia, she examines to what extent their position as outsiders to successful aging enables them to abandon societal guiding ideals and undermine hegemonic-dominant notions of aging. Thus, this paper provides a pertinent example of queering aging beyond a focus on gender and sexuality, and critically reflects on essentialised ideals of successful aging.

Willis et al. consider the housing needs of older people who identify as lesbian, gay, bisexual, and trans (LGBT). Through a queer gerontological lens, the authors examine how older LGBT people are socially situated within mainstream housing schemes where they experience partial visibility while also encountering exclusionary pressures that locate them as “the other.”

Rosenfeld and Ramirez-Valles demonstrate the importance of including life course theory in gay male aging studies, specifically, its focus on cohort membership's implications for later life, including cumulative disadvantage, in addition to more generationally-focused investigations. The authors caution against relying on the field's current exclusive emphasis on identity and individual biography. The analytical power of the life course perspective and queer theory, they argue, lies on their interplay between structure and agency, and between socio-political contexts and LGBTQI^*^ biographies.

These papers critically unpack the application of queer theory in aging research, revealing counter narratives of aging that transgress the boundaries of normative life course models and successful aging. They disturb and challenge many of the norms and understandings that shape and constrain older LGBTQI^*^ people's lives. In doing so, the Research Topic contributes to the advancement of queer aging and LGBTQI^*^ studies, and introduces this emergent yet important theoretical approach to aging studies at large.

## Author contributions

ML: Writing – original draft, Writing – review & editing. DR: Writing – review & editing. RL: Writing – review & editing. JR-V: Writing – review & editing.

## References

[B1] Butler J. (1990). Gender Trouble. Feminism and the Subversion of Identity. New York, NY: Routledge.

[B2] Fabbre V. (2015). Gender transitions in later life: a queer perspective on successful ageing. Gerontologist 55, 144–153. 10.1093/geront/gnu079 25161264 PMC4542896

[B3] Hark S. (2001). Disputed territory: feminist studies in Germany and its queer discontents. Amerika Studien 46, 87–104. 10.25595/326

[B4] Leontowitsch M. Werny R. (2021). “Geschlechter- und Genderforschung in der Soziologie des Alter(n)s: Vom Altweibersommer zu Queeer Ageing,” in Handbuch Soziologie des Alter(n)s, eds. K. R. Schroete, C. Vogel and H. Künemund (Berlin: Springer Reference), 12.

[B5] Lottmann R. (2021). “Altern und Diversität,” in Handbuch Soziologie des Alter(n)s, eds. K. R. Schroeter, C. Vogel and H. Künemund (Berlin: Springer Reference), 40.

[B6] Port C. (2012). No Future? Aging, Temporality, History, and Reverse Chronologies. Interdisciplinary Studies in the Humanities, 4. Available online at: https://shc.stanford.edu/sites/default/files/2012-06/OCCASION_v04_Port_053112_0.pdf

[B7] Ramirez-Valles J. (2016). Queer Aging: The Gaby Boomers and the New Frontier for Gerontology. Oxford: Oxford University Press.

[B8] Rauchut F. (2008). Wie Queer Ist Queer? Sprachphilosophische Reflexionen zur deutschsprachigen akademischen “Queer”-Debatte. Sulzbach: Ulrike Helmer Verlag.

[B9] Rosenfeld D. (2010). “Lesbian, gay, bisexual, and transgender ageing: shattering myths, capturing lives,” in The SAGE Handbook of Social Gerontology, eds. D. Dannefer and C. Phillipson (Thousand Oaks, CA: SAGE Publications Ltd), 226–238.

[B10] Sandberg L. (2008). The Old, the Ugly and the Queer: thinking old age in relation to queer theory. Grad. J. Soc. Sci. 5, 117–139.

[B11] Sandberg L. Marshall B. (2017). Queering aging futures. Societies 7:21. 10.3390/soc7030021

[B12] Sedgwick E. K. (1990). Epistemology of the Closet. A Centennial Book. Berkeley, CA: University of California Press.

